# Incorporation of Edible Plant Extracts as Natural Food Preservatives: Green Extraction Methods, Antibacterial Mechanisms and Applications

**DOI:** 10.3390/foods14234000

**Published:** 2025-11-22

**Authors:** Zafeiria Lemoni, Konstantinos Evangeliou, Theopisti Lymperopoulou, Diomi Mamma

**Affiliations:** 1 Biotechnology Laboratory, School of Chemical Engineering, National Technical University of Athens, Zografou Campus, 9 Iroon Polytechniou Str, 15780 Athens, Greece; zlemoni@chemeng.ntua.gr (Z.L.); kevangeliou@mail.ntua.gr (K.E.); 2 Products and Operations Quality Control Laboratory, School of Chemical Engineering, National Technical University of Athens, Zografou Campus, 9 Iroon Polytechniou Str,15780 Athens, Greece; veralyb@chemeng.ntua.gr

**Keywords:** edible plants, natural preservatives, antibacterial activity, green extraction methods, foodborne pathogens, antibacterial mechanisms, applications

## Abstract

The review article critically evaluates the application of edible plant extracts as natural preservatives in food systems, with a particular focus on environmentally sustainable extraction methodologies. It examines green extraction methods designed to enhance the yield of bioactive compounds responsible for plants’ strong antibacterial properties. The biochemical mechanisms underlying antibacterial activity are studied, namely disruption of bacterial cell walls and membranes; inhibition of metabolic enzymes; interference with nucleic acid synthesis; induction of oxidative stress; and suppression of quorum sensing, biofilm formation, efflux pumps, and β-lactamase activity, along with standardized methodologies for efficacy assessment and extracts’ incorporation into food matrices. Recent research demonstrates the potential of plant extracts to extend the shelf life of meat, seafood, dairy, and fresh products while meeting consumer demand for clean-label products. Although large-scale application remains limited due to challenges, future research should focus on optimizing green extraction approaches, establishing standardized evaluation protocols, and developing regulatory frameworks to facilitate their safe and sustainable use in the food industry.

## 1. Introduction

Antimicrobial resistance (AMR) has emerged as one of the most pressing global health challenges, threatening the efficacy of conventional treatments and compromising food safety and public health. In 2019, AMR was directly responsible for 1.27 million deaths globally, with an additional 3.7 million deaths associated with resistant infections [1]. The overuse and misuse of antibiotics in clinical settings, agriculture, and food production are major drivers of this crisis. In addition, the World Bank estimates that AMR could result in significant economic costs, potentially resulting in US$1 trillion in healthcare costs by 2050 [2]. Simultaneously, consumer demand for safer, “clean-label” food products has intensified the search for alternative antimicrobial solutions [3]. In this context, plants, with their diverse secondary metabolites, have emerged as a promising source of natural antimicrobial compounds.

The demand for natural antimicrobials is especially critical in the food industry, where microbial contamination remains a major cause of spoilage, foodborne illness, and economic loss. Pathogens such as *Escherichia coli*, *Salmonella enterica*, *Listeria monocytogenes*, and *Staphylococcus aureus* continue to pose significant challenges to food safety. These plant-derived compounds can reduce pathogenic loads, extend shelf life, and improve overall food safety [4]. Plants synthesize a wide range of secondary metabolites, such as flavonoids, phenolic acids, tannins, alkaloids, and anthocyanins that offer defense against pathogens. Unlike conventional antibiotics, which typically target a single cellular process (e.g., cell wall synthesis or protein production), plant-derived compounds often act via multiple, overlapping mechanisms [5]. These include disruption of microbial membranes, leakage of cytoplasmic contents, inhibition of essential metabolic enzymes, interference with quorum sensing and biofilm formation, chelation of vital ions, and even direct damage to genetic material [6]. This multi-targeted activity reduces the likelihood of resistance development, positioning plant-derived compounds as possible alternatives to conventional antimicrobials [7]. The antimicrobial effectiveness of plant extracts is determined not only by the specific type of microorganism but also by the extract’s phytochemical composition, which is itself influenced by the extraction method used.

Extraction is a significant step in releasing the antimicrobial compounds of plant matrices, as they are often bound within the plant cell walls or associated with other macromolecules, necessitating their release. Conventional extraction methods, like maceration and Soxhlet, depend on prolonged extraction times, the use of organic solvents, and high energy requirements. Consequently, they are often time-consuming, less selective, and environmentally unsustainable. On the contrary, non-conventional extraction methods, namely Ultrasound-assisted extraction (UAE), Microwave-assisted extraction (MAE), Enzyme-assisted extraction (EAE), and Natural Deep Eutectic Solvents-assisted extraction (NADES), offer higher yield and quality of the final product, along with better preservation of thermolabile compounds [8,9]. Therefore, careful selection and optimization of extraction methods are essential to maximize recovery, activity, and sustainability.

The term “edible plants” refers to plant species that are commonly consumed or used in food preparation, including herbs, spices, fruits, and vegetables, and that are generally recognized as safe for human consumption. It excludes toxic or non-edible species, such as *Atropa belladonna* (deadly nightshade) or *Ricinus communis* (castor bean plant), since they contain tropane alkaloids or ricin respectively, which are highly toxic to humans [10,11]. These edible plants serve as valuable natural sources of bioactive compounds with demonstrated antibacterial potential. It is important to note that this review focuses exclusively on non-volatile plant extracts obtained via environmentally sustainable extraction methods. Essential oils, although derived from many of the same edible plants, are concentrated, volatile liquids produced through methods, such as steam distillation or cold pressing. In contrast, plant extracts are more diluted and contain a wider range of compounds, including non-aromatic ones, extracted by soaking plant material in a liquid solvent. The main difference lies in the extraction method, leading to essential oils being more potent and volatile, and extracts being broader-spectrum and easier to produce. Essential oils’ extraction techniques, chemical nature, and antimicrobial mechanisms have been extensively reviewed [12,13,14,15].

The aim of this review is to critically evaluate edible plant extracts obtained through green extraction technologies as natural food preservatives. We examine their antibacterial mechanisms, summarize the in vitro assays commonly used to assess antimicrobial activity, and discuss how environmentally friendly extraction methods influence the antibacterial efficacy of non-volatile plant-derived bioactive compounds against foodborne pathogens. To maintain a concise and contemporary scope, the review emphasizes edible plant species and literature from the past decade, highlighting recent advances in sustainable extraction strategies. Additionally, the incorporation of plant extracts into food matrices and the assessment of their antibacterial activity within real food systems are discussed, with particular emphasis on future perspectives and the potential for industrial application of these extracts in commercial products.

## 2. Green Extraction Methods for Bioactive-Rich Plant Extracts

Bioactive compounds in plants are commonly classified into three major groups: (a) phenolic compounds, (b) terpenes and terpenoids, and (c) nitrogen-containing compounds [16]. The classification is based on their biosynthetic pathways, namely the shikimic acid (SA) pathway, the malonic acid pathway, the mevalonic acid (MVA) pathway, and the non-mevalonate (MEP) pathway, which determine the chemical structure of the resulting metabolites [17]. Phenolic compounds (PCs) are broadly classified into phenolic acids, flavonoids, tannins, lignans, and stilbenes [18]. Phenolic acids, the simplest PCs, consist of a phenolic ring and at least one organic carboxylic acid group. Based on the number of carboxylic acids, they occur as derivatives of benzoic acid (hydroxybenzoic acids, C6-C1) and derivatives of cinnamic acid (hydroxycinnamic acids, C6-C3) [19]. Flavonoids share a C6-C3-C6 backbone with two aromatic rings (A and B) connected by a heterocyclic chroman ring (C). Structural variations namely, substitution pattern of the C-ring, number and position of hydroxyl, methoxyl, or glycosidic groups on the A and B rings, give rise to flavonoid subclasses, including flavones, flavonols, flavanones, flavanols, isoflavones, and anthocyanidins [20]. Tannins include hydrolysable tannins, composed of gallic or ellagic acid esters, and condensed tannins (proanthocyanidins), which are oligomers or polymers of flavan-3-ol units [21]. Lignans consist of two phenylpropanoid dimers linked by a carbon-carbon bond, while stilbenes consist of two benzene rings connected with C2-C6 with a double bond [20]. Terpenes are characterized by a hydrocarbon structure, while terpenoids are modified forms of terpenes, with various functional groups and oxidized methyl groups at different positions [22]. Nitrogen-containing compounds, mainly alkaloids, are cyclic compounds that consist of carbon and nitrogen atoms. The basic nitrogen atom can occur in the form of primary amine (RNH_2_), secondary amine (R_2_NH), or tertiary amine (R_3_N) [23].

Plant-derived bioactive compounds are often trapped within the complex plant cell wall matrix, making their extraction a challenging process [24]. Extraction methods are broadly divided into conventional, such as Soxhlet extraction, maceration, hydro-distillation, and cold pressing, and non-conventional or “green” methods, including UAE, MAE, EAE, and green solvents-assisted extraction, like NADES [25]. Conventional methods have historically served as the backbone of natural product extraction, relying on prolonged contact between plant material and solvents, often at elevated temperatures [26]. For instance, Soxhlet extraction continuously recirculates hot solvent, while maceration and percolation achieve solubilization of secondary metabolites through extended solvent contact [27]. Mechanical approaches, such as pressing or expellers, can also disrupt plant structures, though they often degrade compounds by breaking molecular chains, leading to reduced yield and bioactivity [28]. Chemical methods, in contrast, depend on organic solvents to penetrate the plant cell wall and release compounds [29]. While effective, these processes present significant drawbacks: high energy demand, time-intensive operation, risk of thermolabile compound degradation, use of large volumes of toxic solvents, and concerns over residual toxicity in extracts [29]. Despite these shortcomings, conventional methods remain important, providing reference points for yield, selectivity, and bioactivity when evaluating novel technologies. However, the limitations of these techniques have driven the development of greener, more efficient extraction strategies.

### 2.1. Green Solvents

Extraction methods have evolved significantly in recent years, with new, cheaper, faster, and mostly greener methods trying to replace the conventional ones. Green technology is highly focused on creating new solvents with safer ecotoxicological profiles, cheaper prices, and desired qualities for various technological processes. The beginning of this effort was made with ionic liquids (ILs), which received a lot of interest in the context of replacing toxic organic solvents [30]. Despite their explicit properties, they are not as environmentally friendly as it was intended to be, leading to the discovery of a new class of green solvents, Deep Eutectic Solvents (DES) by Abbott et al. [31,32,33]. DESs are a mixture of a hydrogen bond acceptor (HBA) and a hydrogen bond donor (HBD), with melting points lower than the melting points of the individual components [31]. Compared to ILs, DES are superior, due to the low cost of their raw materials, the simplicity of manufacturing procedures, the pharmaceutically acceptable toxicity, and high biodegradability [34,35]. Although DES demonstrated a significant improvement compared to organic solvents, they were not completely free of toxic components. Choi et al. created a subclass of DES, whose individual components are metabolites of natural origin, and named them NADES [36]. The main composition is the same as DES, and the ingredients are sugars, carboxylic acids, amino acids, choline, and water. Since then, more than 150 NADES have been composed. NADES are less expensive, completely sustainable, non-toxic, biodegradable, and more eco-friendly compared to ILs and DESs. Hence, they are perfectly embodying the green chemistry principles [37,38]. Furthermore, they have demonstrated the ability to disintegrate macromolecules, suggesting that they have a high potential for use as solvents in the extraction of secondary metabolites for use in the food or pharmaceutical industries [39].

### 2.2. Ultrasound-Assisted Extraction

Ultrasound-assisted extraction is a green technology that uses high-frequency sound waves (>20 Hz) to extract natural products from plant matrices. These waves comprise a sequence of compression and rarefaction cycles that can penetrate solid, liquid, or gaseous media [40]. At elevated sound wave intensities, the negative pressure during rarefaction surpasses the cohesive forces between molecules, resulting in their separation and the formation of cavitation bubbles [41]. The bubbles expand through coalescence and subsequently collapse during the compression phase, generating fragmentation, localized erosion, and enhanced absorption [41]. The cavitation bubbles create mechanical and thermal effects on the plant cell walls, leading to their degradation, and finally the release of the bioactive compounds into the solvent through diffusion or dissolution. UAE has been shown to enhance the yield of bioactive compounds compared to conventional methods, while also significantly reducing extraction time [42]. The key parameters influencing UAE are ultrasonic power, extraction temperature, and extraction time. These factors should be tailored in accordance with the target compounds to maintain their stability, as UAE can produce intense conditions (temperature and pressure) [40,43].

### 2.3. Microwave-Assisted Extraction

The mechanism of MAE relies on the ability of microwaves to penetrate the plant matrix. Microwaves are high-frequency electromagnetic waves between infrared waves and radio waves in the electromagnetic spectrum (frequency 0.3–300 GHz) [44]. The electromagnetic waves heat the moisture inside the cells, evaporate it, and eventually produce a high pressure which causes changes in the cell structure. In this way, the porosity of the cell matrix increases, allowing better penetration of the solvent through the matrix and improved yield of the desired compounds [45]. In the microwave process, the energy is transferred by two mechanisms: dipole rotation and ionic conduction. The dipole rotation is responsible for the rearrangement of the dipoles within the applied field. The ionic conduction is the electrophoretic migration of ions when an electromagnetic field is applied, whereas the resistance of the solution to this flow of ions produces the friction that heats the solution [46,47]. Although in conventional extraction methods the energy is transferred by convection, conduction, and radiation phenomena through the external material surface, in MAE, the microwave energy is transferred directly to materials through molecular interactions via conversions of electromagnetic energy into thermal energy [46]. MAE requires small amounts of solvents and reduces the extraction time, resulting in a lower environmental impact. However, several drawbacks come with MAE, such as the high cost of the equipment and the requirement of organic solvents, when water is not efficient enough [44].

### 2.4. Enzyme-Assisted Extraction

Enzyme-Assisted Extraction is an advanced technique that utilizes the natural ability of enzymes to selectively break down the complex macromolecular structure of plant cell walls, thereby facilitating the release of target bioactive compounds. Due to the structural complexity of plant tissues, EAE often requires the combined action of multiple enzyme groups, along with appropriate pretreatment steps and careful control of process parameters to optimize extraction efficiency and yield [48]. By acting on polysaccharide and lignin matrices, enzymes enhance cell wall permeability, allowing easier solvent penetration and improved recovery of bioactive constituents [28]. The selection of enzymes is critical in EAE and should be guided by the compositional analysis of the plant cell wall, ensuring that the selected enzymes effectively target its major structural components for maximal disruption and compound release [49]. If necessary, a combination of enzymes could be applied to achieve a more efficient degradation of the plant cell wall, since composition can vary even within the same species [50]. The most commonly used enzymes include cellulases, hemicellulases, pectinases, and proteolytic enzymes. EAE offers an eco-friendly and more selective alternative to conventional methods, minimizing solvent consumption and reducing environmental footprint [51].

### 2.5. Supercritical Fluid Extraction

Supercritical fluid extraction (SFE) is an alternative extraction system employed for the selective extraction of bioactive compounds from medicinal plants, utilizing fluids above their critical temperature and pressure. The most commonly used supercritical fluid is carbon dioxide (CO_2_) due to its low critical point (31.1 °C, 73.8 bar), non-toxic nature, and ease of removal after extraction. In the supercritical state, CO_2_ exhibits gas-like diffusivity and liquid-like solvating power, allowing efficient penetration into plant cell walls and dissolution of target compounds, especially low-polar compounds (MW under 250) [52]. The addition of polar co-solvents (modifiers), such as ethanol, methanol, and water, enhances the solubility of highly polar bioactive compounds (MW over 400) that cannot be easily dissolved in supercritical carbon dioxide [52]. SFE provides several advantages, including high selectivity, minimal thermal degradation of thermo-sensitive compounds, solvent-free final products, and reduced environmental impact. It is particularly effective for isolating bioactive compounds, while preserving their quality and biological activity [53].

### 2.6. High Hydrostatic Pressure

High hydrostatic pressure (HHP) is an emerging technology that leverages Le Chatelier’s principle, applying high pressure to a matrix, transmitted by a liquid in a closed system [54]. High pressure induces phenomena like phase transitions, altered reaction dynamics, and molecular structure changes, enhancing extraction efficiency [55]. HHP changes the conformation or denatures cell membrane proteins, making them less selective and thus making the bioactive compounds more accessible for extraction [55]. Based on phase behavior theory, the dissolution is faster at higher pressure, because under the large differential pressure created between the interior and the exterior of the cell, the solvent penetrates the membrane, and the mass transfer rate of solute or the rate of dissolution increases [54,56]. This process reduces mass transfer resistance within the cell, facilitating the release of bioactive components while others remain intact [56]. HHP requires short incubation times, low temperatures (up to 60 °C) at high pressures (100–600 MPa), preventing the thermal degradation of thermosensitive compounds [55,56].

### 2.7. Polarity and Extraction Compatibility

The effectiveness of green extraction methods is strongly influenced by the match between solvent and target compound polarity, combined with the physical enhancement mechanisms provided by each green extraction method. The major classes of bioactive compounds differ markedly in chemical structure and thus in polarity and solubility. Phenolic compounds are generally polar due to multiple hydroxyl groups attached to the aromatic ring and are therefore readily soluble in polar solvents such as water, ethanol, methanol, or hydroalcoholic mixtures, although solubility decreases for high-molecular-weight phenolics [57]. Terpenes, in contrast, are highly non-polar hydrocarbons and are insoluble in water but soluble in non-polar organic solvents (e.g., hexane, chloroform), whereas terpenoids show variable polarity depending on the presence of oxygenated functional groups, with some highly polar glycosylated terpenoids becoming water-soluble [58]. Nitrogen-containing compounds, such as amines and alkaloids, are generally polar and water-soluble due to hydrogen-bonding, though solubility decreases as non-polar alkyl chain length increases. The presence of oxygen functional groups on nitrogen compounds increases their polarity, making them more susceptible to extraction with polar solvents [59].

Therefore, each extraction method could be suited to a specific target compound (Table 1). UAE and MAE combined with polar solvents are highly effective for phenolics, whereas their application to non-polar terpenes requires non-polar solvents [43,45]. EAE is particularly useful for phenolics and certain alkaloids that are physically trapped within the cell wall matrix [51]. NADES provide a versatile green alternative, as their polarity and hydrogen-bonding capacity can be tailored to selectively extract phenolics, terpenoids, or nitrogen-containing compounds [39]. SFE with CO_2_ is well suited for non-polar compounds, particularly terpenes and terpenoids, although it has been used with cosolvents to extract all categories of metabolites [60]. Therefore, these highlight that the physicochemical characteristics of bioactive compounds must guide solvent choice and extraction technology selection.

### 2.8. Comparative Evaluation of Green Extraction Technologies

A thorough comparison of green extraction methods shows that they differ substantially in several aspects namely, extraction yield, solvent requirements, energy demand, operational limitations, and industrial application. As outlined in Table 1, NADES-based extraction is an emerging method, which provides high efficiency but is constrained by issues such as high viscosity and difficulties in large-scale solvent recovery. MAE and UAE generally exhibit high yields with low energy consumption and minimal solvent use, making them attractive for sustainable processing in several industries, and their scalability is constrained. EAE and SFE are both well established and already widely accepted in food and pharmaceutical sectors despite their high enzyme and capital cost, respectively. HHP also demonstrates strong scalability and regulatory approval but remains limited by its batch nature. Across all methods, challenges persist regarding compound stability, equipment investment, and operational expertise. For industrial implementation, future directions should focus on developing efficient recovery and purification strategies for green solvents such as NADES, designing continuous or hybrid extraction systems to improve throughput, and establishing standardized quality and safety frameworks to support regulatory acceptance. Enhanced real-time monitoring, extract standardization, and rigorous toxicological evaluation will be essential to fully integrate these sustainable extraction methods into large-scale manufacturing [61,62,63].

## 3. Antibacterial Assays

The assessment of antibacterial activity of plant-derived extracts is a critical step in validating their potential as food preservatives. Plant extracts are chemically complex, containing multiple bioactive molecules that may act via diverse mechanisms, including disruption of microbial membranes, enzyme inhibition, nucleic acid interactions, oxidative stress induction, metal chelation, quorum sensing interference, and synergistic enhancement with other antimicrobials. The selection of a method is based on many factors, such as flexibility, automation, cost, reproducibility, and accuracy. A combination of in vitro assays is typically employed to comprehensively evaluate the antibacterial potential of plant extracts, elucidate dose–response relationships, and correlate mechanistic effects with functional outcomes [7,64].

### 3.1. Agar Diffusion Assays

Agar-based diffusion methods, including the disk diffusion and well diffusion assays, are among the most widely used initial screens for plant extract antibacterial activity. In disk diffusion, sterile filter paper disks impregnated with known concentrations of extracts are placed on agar plates inoculated with target microorganisms, whereas in well diffusion assays, the wells are bored into agar and filled with extracts. During incubation at a predetermined temperature and time, depending on the microorganism, the extract diffuses into the agar, creating a concentration gradient. If the extract possesses antibacterial activity, it inhibits the growth of the surrounding microorganisms, resulting in a clear zone around the disk or well, providing a qualitative estimate of antibacterial activity. Both techniques allow comparison between extracts and standard antibiotics and can be used for both bacteria and fungi [65,66]. However, these assays are limited by factors such as extract solubility, diffusion rate, and molecular size, providing only preliminary indications of activity rather than precise quantitative data. They are unsuitable for determining minimum inhibitory concentration (MIC) values, as non-polar compounds may not diffuse well from a disk, and results can lack reproducibility, particularly with crude extracts [65,67].

### 3.2. Broth Dilution Assays

Broth dilution and microdilution are standard antibacterial susceptibility tests used for the determination of MIC and the Minimum Bactericidal Concentration (MBC). The MIC is the lowest concentration of an antibacterial that prevents visible bacterial growth, whereas the MBC is the lowest concentration required to kill the microorganism, typically defined as achieving a 99.9% reduction in viable cells. MIC tests are typically performed first and then followed by MBC tests. The classic broth macrodilution method uses test tubes, while microdilution is a more efficient version performed in multi-well plates. The antibacterial is serially diluted in a liquid growth medium (usually in two-fold dilutions), then a standardized suspension of the microorganisms is added, then the plates or tubes are incubated under appropriate conditions for the specific microorganism (typically 12–24 h) and the growth is measured either via optical density or colorimetric indicators such as resazurin, tetrazolium salts, or INT (iodonitrotetrazolium) [65,68]. After the MIC test is complete, the samples from the wells that showed no visible growth (above the MIC) are transferred to a new medium without the antibacterial to determine if the antibacterial is bacteriostatic (inhibits growth) or bactericidal (kills bacteria) [69,70,71].

### 3.3. Time-Kill Kinetics

Time-kill assays are an effective method for providing information about the dynamic interaction between the antibacterial agent and the microbial strain. The time-kill test reveals a time-dependent or a concentration-dependent antibacterial effect. The cultures are incubated under controlled conditions, samples are taken at predetermined time intervals, and the number of viable microorganisms in each sample is determined at each time point. The most common method is to use serial dilution and spread plating to count colony-forming units (CFUs) on agar plates. The data from the different time points are used to construct a time-kill curve, which illustrates how the microbial population changes over time. The assay not only reveals the rate at which the extract kills microbes, but can distinguish between a bactericidal (killing) and bacteriostatic (inhibiting growth) effect by measuring the reduction in viable cells [65,72].

### 3.4. Thin-Layer Chromatography Bioautography

Thin-layer chromatography (TLC) bioautography is a rapid and effective method for detecting antibacterial substances directly on chromatographic plates. The test samples are separated on TLC plates, which are then exposed to bacterial cultures. The addition of tetrazolium salts such as MTT, TTC, or INT allows visualization of bacterial activity, as living cells convert these salts into colored formazans, creating clear inhibition zones around active compounds. Modern approaches combine TLC bioautography with high-performance liquid chromatography (HPLC) or over-pressured-layer chromatography (OPLC) for identification of active agents. This integrated approach has been successfully applied to plant extracts and is expected to gain increasing importance in future antibacterial research [72,73].

### 3.5. Antibiofilm Assay

The antibiofilm activity of plant extracts is most commonly assessed using the microtiter plate crystal violet (CV) assay. This method quantifies biofilm biomass by staining surface-adherent cells with CV and measuring the retained dye spectrophotometrically. A reduction in absorbance indicates inhibition or disruption of biofilm formation. This method provides a simple, reproducible, and quantitative way to compare antibiofilm effects of plant extracts [74,75,76].

### 3.6. Flow Cytometry Assay

Flow cytometry provides rapid and sensitive evaluation of the antibacterial activity of extracts by measuring cell viability, membrane integrity, and metabolic activity using fluorescent dyes. Dyes such as propidium iodide (PI) and cFDA indicate cell damage through membrane depolarization. Stained cells are then analyzed using a flow cytometer, and the resulting data allow quantification of viable, injured, and dead cells, providing a detailed understanding of the extract’s antibacterial effects relative to controls. Unlike agar diffusion and dilution methods, flow cytometry quantifies sub-lethally stressed or non-culturable cells, and discriminates different cell populations, enabling high-throughput assessment of antimicrobial efficacy [77,78,79].

### 3.7. Plate Count Methods

Standard Plate Count (STC), often referred to as Total Viable Count (TVC), Aerobic Colony Count (ACC), or Aerobic Mesophilic Count (AMC), is the most widely used technique for microbiological quality assessment in food products. It quantifies the total population of microorganisms capable of growing under aerobic conditions and moderate temperatures present in a sample at the time of testing. Typically, the food matrix is homogenized (e.g., by stomaching or blending), serially diluted to the appropriate concentration, and mixed with nutrient-rich agar before incubation at a controlled temperature (commonly 30 °C). The resulting visible colonies are enumerated as Colony Forming Units per gram or milliliter (CFU·g^−1^ or CFU·mL^−1^) [80]. Selective plating methods have been developed for the detection and subsequent enumeration of most known foodborne pathogens such as Coliforms, *Enterobacteriaceae*, *E. coli*, various *Salmonella* spp., yeasts and molds, as well as psychrophilic bacteria [81]. Foods treated with plant extracts are frequently analyzed using TVC to evaluate background microbiota before and after treatment, as well as during storage, enabling the assessment of microbial reduction (CFU·g^−1^ change) relative to control samples. Regulatory agencies (e.g., European Commission, EU; United States Food and Drug Administration, FDA; UK Health Security Agency, UKHSA) publish guidelines limits for acceptable TVC in various food categories with raw and fermented foods, typically presenting higher counts (10^6^ to 10^7^ CFU·g^−1^) than heat-treated products (<10^3^ to 10^4^ CFU·g^−1^) [82,83].

### 3.8. Microbiome Profiling

Next-Generation Sequencing (NGS) offers a detailed and comprehensive assessment of the microbial community in a food matrix. It involves 16S rRNA gene sequencing, using platforms like Illumina MiSeq to characterize the total microbial ecology. It involves the extraction of V4–V5 region of the bacteria’s 16S ribosomal RNA gene from a food sample, utilizing manual or automated systems and subsequent amplification through the use of Polymerase Chain Reaction (PCR) and the appropriate set of primers. The resulting amplicons are extracted from the agarose gel, purified, and then subjected to sequencing. This technique, when performed in time intervals, reveals the progress of growth of certain target microorganisms or groups during the storage process, providing more detailed insight than plating methods [81,84].

## 4. Antibacterial Activity of Plant Extracts Against Foodborne Pathogen Microorganisms

Extracts of edible plants are increasingly studied as potential alternatives or complements to synthetic antimicrobials due to being a rich source of bioactive compounds. However, the antibacterial potential of these extracts largely depends on both the phytochemical profile achieved and the interaction between secondary metabolites and microbial cell structures. Extensive research has explored the antibacterial activity of various plant matrices and their correlation with specific bioactive compounds to enhance their efficacy. While most studies focus on conventional extraction methods, green extraction techniques have emerged as effective approaches to obtain higher-quality and higher-yield extracts (Table 2).

Gonelimali et al. [64] compared conventional heating and UAE to obtain ethanolic and aqueous extracts from roselle (*Hibiscus sabdariffa*), rosemary (*Rosmarinus officinalis*), clove (*Syzygium aromaticum*), and thyme (*Thymus vulgaris*). UAE consistently produced higher extract yields and enhanced antibacterial potency. Ethanolic and aqueous extracts of roselle and clove inhibited the growth of both Gram-positive (*Bacillus cereus*, *Staphylococcus aureus*) and Gram-negative (*Escherichia coli*, *Salmonella* Enteritidis, *Vibrio parahaemolyticus*, and *Pseudomonas aeruginosa*) bacteria, while the fungus *Candida albicans* responded only to clove and thyme ethanolic extracts. The potential antibacterial mechanism of the above plant extracts involves cytoplasmic acidification and cell wall disruption [64]. Experimental studies applying UAE of Finnish plant leaves demonstrated strong antibacterial activity against Gram-positive bacteria. Leaf extracts from lingonberry, saskatoon, and sea-buckthorn exhibited over 90% inhibition of *S. aureus*, *B. cereus*, and *L. monocytogenes*, with sea-buckthorn showing near-complete inhibition of *S. aureus* and *B. cereus*. In contrast, the Gram-negative bacterium *E. coli* was largely resistant, while *S. enterica* exhibited intermediate susceptibility. Statistical analysis revealed a strong correlation between ellagitannin content and inhibition of *S. aureus* and *B. cereus*, and between proanthocyanidins and inhibition of *L. monocytogenes*. These results support the hypothesis that the higher number of hydroxyl groups in ellagitannins and proanthocyanidins enhances disruption of Gram-positive bacterial cell walls, explaining the observed selectivity [85]. Criste et al. [86] sonicated berries and leaves of *Hippophae rhamnoides* L. and measured the antibacterial activity against *S. aureus*, *B. cereus*, and *P. aeruginosa* using a broth-microdilution MIC method. Leaf extracts consistently exhibited stronger antibacterial activity than berries, a difference attributed to the higher polyphenol content, especially gallic acid and quercitrin (quercetin 3-rhamnoside), which were the most abundant phenolics in all leaf extracts. Similarly, rosemary leaves extracted by UAE demonstrated significantly enhanced antibacterial activity compared to conventional extraction. The UAE extract was particularly effective against Gram-positive *S. aureus* (MIC 0.14 mg/mL), while *P. aeruginosa* exhibited the highest resistance. MICs were reduced by up to 60% relative to the conventional extract. The antibacterial activity was attributed to phenolic compounds, primarily rosmarinic acid, carnosic acid, and carnosol [87]. UAE was also applied to three cultivars of *Chaenomeles japonica* (Japanese quince) fruit to evaluate their antibacterial properties. All cultivars exhibited inhibitory effects against three Gram-positive and three Gram-negative bacterial strains, with *Enterococcus faecalis* (ATCC 29212) showing the highest sensitivity; however, no activity was observed against the yeast *Candida albicans*. The antibacterial activity of the extracts correlated strongly with their rutin content (radius of the inhibition zones r = 0.98, 0.94, 0.92, 0.69, and 0.69 mm for *S. aureus*, *E. coli*, *E. faecalis*, *B. subtilis*, and *P. aeruginosa*, respectively) and with epicatechin levels (r = 0.94 mm for *Salmonella* spp.) [88]. Although, UAE of *Rosa canina* L. fruit yielded extract with mild to poor activity (MIC 256–512 mg L^−1^) against multidrug-resistant (MDR) bacterial strains (*Staphylococcus aureus* SA1199B, and EMRSA16) it was able to enhance the effectiveness of tetracycline against the tetracycline-resistant strain *S. aureus* XU212, which carries a multidrug efflux mechanism and also showed a moderate inhibitory effect on plasmid conjugation. The authors suggested that the observed antibacterial activity is not due to inherent bactericidal effects, but rather results from disruption of efflux pumps and plasmid transfer, indicating potential resistance-modulating properties [89]. Pomegranate (*Punica granatum* L.) peels have been extensively investigated for their antibacterial properties. Peršurić et al. [90] evaluated eight ethanol extracts derived from UAE and reported that. *S. aureus* was the most sensitive (MIC = 0.8–6.4 mg mL^−1^) while *L. monocytogenes* was the least (MIC = 12.8 mg mL^−1^). Overall, extracts displayed broader activity against Gram-positive strains and variable efficacy against Gram-negative ones, attributing the effect generally to the high phenolic/ellagitannin content rather than a single specific compound [90].

Grillo et al. [91] evaluated the antibacterial activity of MAE-derived pomegranate peel extracts and reported that the highest inhibition was observed against *S. aureus*, with inhibition halos exceeding the positive control (chloramphenicol). Other tested pathogens (*E. coli*, *P. aeruginosa*, *S. typhimurium*) showed moderate susceptibility (≈15–19 mm at 500 µg mL^−1^), while *Listeria monocytogenes* and *Bacillus subtilis* were resistant [91]. Similarly, Abutayeh et al. [92] compared the MAE of pomegranate peels with maceration and aqueous extraction. MAE yielded extracts with the strongest activity against *S. aureus* (MIC 12.5 µg µL^−1^), and notable synergism with gentamicin against resistant *P. aeruginosa* (MIC 0.39–3.125 µg µL^−1^) [92]. The authors of both studies concluded that MAE not only enhanced the extraction yield but also preserved the phenolic compounds related to antibacterial activity of the extracts. In the study of Singh et al. [93] antibacterial activity of MAE-derived *Piper betle* L. extract was linked to phytol and neophytadiene, both of which have been previously reported to exhibit antibacterial properties. The extract demonstrated measurable zones of inhibition against all bacterial strains tested, with the most pronounced effects observed against *B. pumilus* (3.6 mm at 100 mg mL^−1^) and *E. coli* (3.0 mm). Antibacterial activity of MAE-derived oregano extract was superior to that of lovage against both *E. coli* and *S. aureus*, and that was attributed to high rosmarinic acid content in the oregano extract [94]. Ürgeová et al. [95] reported that MAE-derived hydrolates from five *Salvia* species showed variable antibacterial activity. Hydrolates of *S. officinalis* and *S. sclarea* showed antibacterial effects against all bacteria tested (*E. coli*, *M. luteus*, *E. asburiae,* and *B. subtilis*) while the extracts of *S. sclarea* exhibited stronger inhibition on Gram-positive bacteria. In *Salvia* spp. hydrolates, terpenes such as isopulegol, 1,8-cineole, thujone, borneol, and linalool were predominant, with borneol, thujone, and 1,8-cineole particularly associated with antibacterial effects. Minor constituents like 1,8-cineole may further enhance antibacterial activity, while phenolics such as eugenol can inhibit microbial enzymes through interactions with protein hydroxyl groups. *Olea europea* leaves were extracted by applying MAE with water, ethanol, and glycerol as solvents, and the resulting extracts were tested against *S. aureus*, *S. typhimurium*, *E. coli*, *L. monocytogenes*, and *Y. enterocolitica*. The water MAE extract showed superior antibacterial activity (MIC 2.5 mg mL^−1^ for *S. aureus*), despite lower total phenolic content compared to ethanolic extracts. Minor phenolics such as hydroxytyrosol, elenolic acid derivatives, and protocatechuic acid were identified as main contributors, acting synergistically to disrupt bacterial membranes and inhibit growth more effectively than the dominant oleuropein [96].

Januskevič et al. [97] compared solid–liquid extraction (SLE) and EAE of *Aralia cordata* leaves and roots, sea buckthorn (*Hippophae rhamnoides*), and hemp (*Cannabis sativa*) leaves. Antibacterial testing against seven pathogens revealed that only sea buckthorn leaf extracts inhibited *S. aureus* (SLE: 2.33 mm; EAE: 4.17 mm). The enhanced activity of EAE extracts was attributed to increased release of bioactive compounds (phenolics, proteins, and sugars) following enzymatic cell wall degradation, as confirmed by SEM imaging showing pronounced structural disruption of plant tissues [97]. In two studies, the EAE of *Rosa canina* L. pseudo-fruit was investigated, employing either a cellulolytic enzyme preparation (Cellic^®^ CTec3 HS) [98] or a combination of a pectinolytic (Pectinex^®^ Ultra Color) and a hemicellulolytic (Viscoferm^®^) enzyme preparation [99]. In the former study, the extract achieved 80% inhibition of *E. coli* growth, while in the latter study, only 55% inhibition of the same bacterium. Alexandre et al. [100] subjected pomegranate (*Punica granatum* L.) peels to high-pressure extraction (HHP, 300 and 600 MPa, 15 min), EAE (4% pectinase plus 4% cellulase, 15 min), and a combination of HHP and EAE. All extracts selectively inhibited pathogenic bacteria, sparing the beneficial ones. HHP at 600 MPa yielded the lowest MIC values for most tested strains, outperforming both EAE and HHP-EAE, except for *E. coli* and *S.* Enteritidis, where all methods showed the same MIC (62.5 mg/mL). Notably, the HHP (600 MPa) extract exhibited particularly low MIC values against *B. cereus* (0.98 mg mL^−1^) and *P. aeruginosa* (31.25 mg mL^−1^). The selective antibacterial effect correlated strongly with phenolic content, especially punicalagin isomers and bis-HHDP-glucoside, indicating that phenolic composition, rather than extraction method alone, affects antibacterial efficacy.

Žitek et al. [101] extracted *Origanum vulgare* by SFE with CO_2_ and compared it to maceration. The SFE extract showed the lowest MICs, indicating stronger antibacterial potency than the macerated extract, with a variation in the MIC values from 0.147 to 2.712 mg/mL. The authors attributed the antibacterial effect to carvacrol and thymol, which are known for strong antibacterial properties.

Two different studies employing NADES-assisted extraction, with 70% ethanol extracts as the reference, consistently reported that the choline chloride:citric acid (1:1, 30% water) NADES was the most effective solvent system. This formulation produced extracts with the strongest antibacterial activity among all tested NADESs. Jurić et al. [102] demonstrated that the NADES-derived extracts of *Mentha piperita* inhibited the growth of all bacteria tested (*P. aeruginosa*, *S. aureus*, *E. coli*, and *Salmonella enterica*) at 0.39–0.78% dilution, significantly lower than the 25% required for the sugar- or alcohol-based NADES and the 70% ethanol extract. Similarly, Memdueva et al. [103] demonstrated that the NADES-derived *Malva sylvestris* L. extracts exhibited strong antibacterial activity against *S. aureus*, *E. coli*, *P. aeruginosa*, and *B. cereus* (inhibition zones ≈ 30–33 mm), comparable to gentamicin (positive control), while ethanol extracts were largely inactive. Moderate antifungal activity was observed against *A. niger* and *P. chrysogenum* (inhibition zones ≈ 10–15 mm). Both studies attributed the superior antibacterial performance of NADES extracts to their low pH, which may promote protein denaturation and membrane disruption, further enhanced by electrostatic interactions between cholinium ions and microbial cell walls. However, Jurić et al. [102] suggested that the antibacterial activity of the extracts was linked strongly to the intrinsic toxicity of the NADES, while Memdueva et al. [103] observed a sharp decrease in acidity after extraction, indicating that the antibacterial activity primarily arose from the bioactive compounds rather than the solvent itself. Further research is therefore needed to elucidate the contributions of NADES components and extracted metabolites to the overall antibacterial activity.

Collectively, the evidence reinforces that each green extraction method preferentially enhances the recovery of specific categories of bioactive compounds, not because the techniques lack versatility, but because their physicochemical principles inherently favor certain molecular classes. Overall, these methods show strong potential for generating food-grade antibacterial extracts suitable for safe application in food preservation; however, further optimization, process standardization, and validation are still needed to fully support their industrial implementation.

## 5. Antibacterial Mechanisms of Plant Extracts

Plant extracts have been evidenced to be valuable sources of bioactive compounds with potent antibacterial properties. Their efficacy arises from a wide variety of phytochemicals, such as phenolics, flavonoids, terpenoids, and alkaloids, that act individually or synergistically to inhibit microbial growth. Unlike conventional antibiotics that often target a single cellular site, plant extracts typically exhibit multifaceted mechanisms of action, making them less prone to induce bacterial resistance. Understanding these mechanisms is crucial for elucidating how plant metabolites exert their antibacterial effects and for guiding the development of novel natural therapeutics or antibiotic adjuvants. The main mechanisms by which plant extracts display antibacterial activity are the disruption of cell membrane and cell wall integrity, the inhibition of enzymes and metabolic pathways, the interaction with nucleic acids and microbial proteins, the inhibition of efflux pumps and degrading enzymes (like β-lactamases), the induction of oxidative stress, and the blockage of quorum sensing and biofilm formation.

### 5.1. Cell Wall Structure of Gram-Positive and Gram-Negative Bacteria

**Figure 1:**
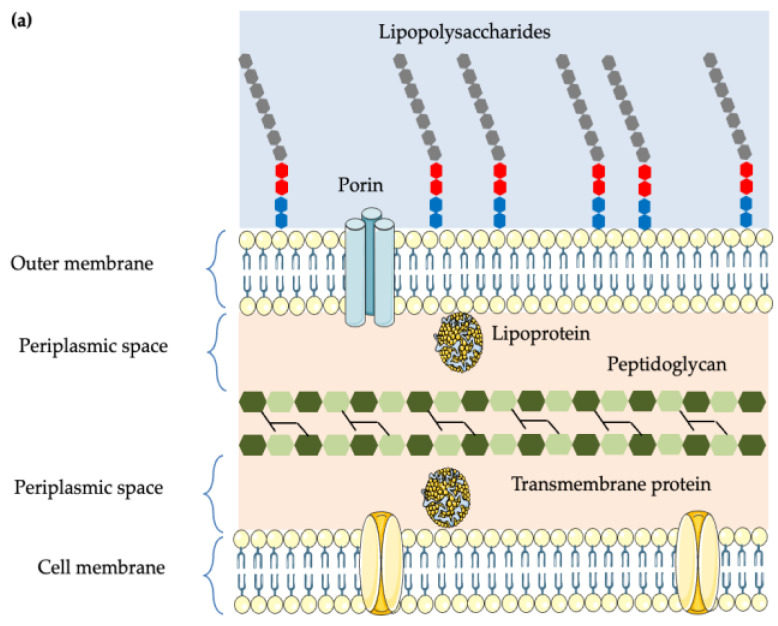

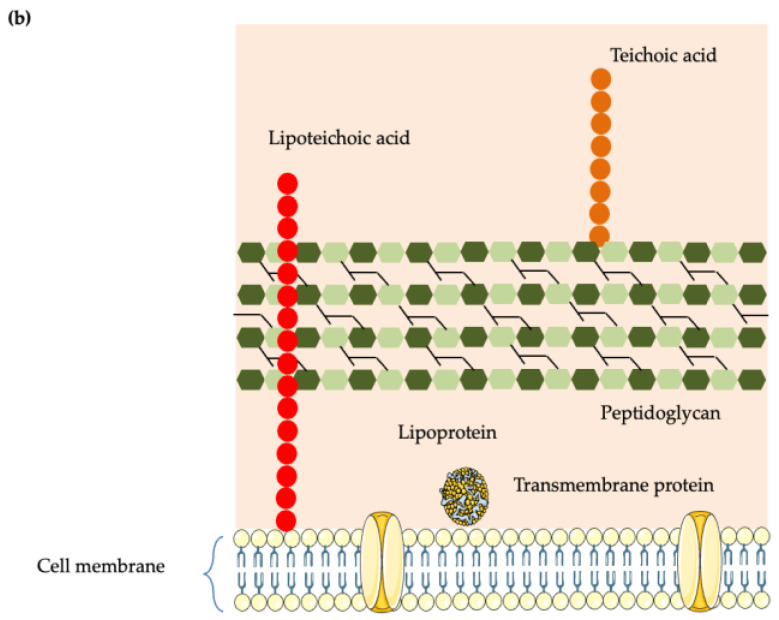
The cell wall structure of Gram-positive (**a**) and Gram-negative (**b**) bacteria.

Gram-negative and Gram-positive bacteria differ fundamentally in their cell wall structure, which strongly influences their antibiotic susceptibility (Figure 1). Gram-negative bacteria possess a complex, three-layered envelope that enhances their survival and resistance in hostile environments. The first layer is the outer membrane, composed of phospholipids in the inner leaflet of the membrane and lipopolysaccharides in the outer leaflet. Moreover, the outer membrane contains proteins called the outer membrane proteins, such as porins, which allow the passage of small molecules, like amino acids and small saccharides [104]. The second layer is the peptidoglycan cell wall, which is a rigid exoskeleton that determines the cell shape and consists of a repeat unit of the disaccharide N-acetylglucosamine-N-acetylmuramic acid [105]. The third layer is the inner membrane, a phospholipid bilayer responsible for multifunctional processes like structure, transport, and biosynthetic functions. In contrast, Gram-positive bacteria lack an outer membrane, but are surrounded by a thick peptidoglycan layer composed of ten to forty layers, whereas Gram-negative bacteria have only one or two thin layers. The peptidoglycan layer provides rigidity and strength and contains teichoic and lipoteichoic acids, which play roles in cell wall maintenance and ion regulation [106]. Overall, this structural distinction not only defines their Gram reaction but also affects their resistance mechanisms. Gram-positive bacteria are structurally simpler, but their lack of the outer membrane makes them more vulnerable to cell wall-targeting antibiotics [107].

### 5.2. Disruption of the Cell Membrane and Cell Wall Integrity

**Figure 2:**
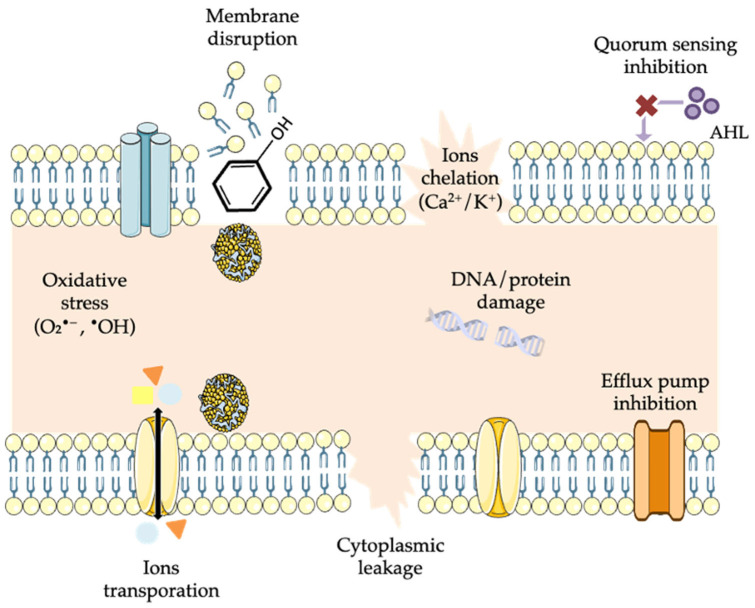
Antibacterial mechanisms of bioactive compounds.

Many studies have identified the disruption of bacterial cell walls and membranes as the main antibacterial mechanism of polyphenols (Figure 2). The bacterial cell wall maintains structural integrity and osmotic balance in both Gram-positive and Gram-negative bacteria, and its damage weakens resistance to external stress. The bacterial cell membrane serves as a selective barrier that controls the transport of nutrients, ions, and waste, while also maintaining energy production and cellular homeostasis [108]. The ability of polyphenols to disrupt bacterial cell walls and membranes is influenced by both their molecular structure and the type of bacterium involved. Polyphenols can directly interact with and damage both bacterial cell wall and membrane, disrupting physiological functions, altering cell morphology, and interfering with metabolism [108,109]. In Gram-negative bacteria, polyphenols accumulate within lipid bilayers, disrupt lipoprotein interactions, and increase membrane permeability, causing leakage of small molecules (e.g., potassium, ATP) along with larger macromolecules (e.g., nucleic acids, proteins) [109,110]. Collectively, these effects result in cell death. For instance, hydroxylic groups in gallic and ferulic acid can bind to peptidoglycans, inducing localized hyper-acidification, pore formation, and subsequent cell death [109,110]. Flavonoids interact with lipid bilayers through two distinct mechanisms depending on their polarity: non-polar flavonoids embedded within the hydrophobic core of the membrane and disrupt its structure, whereas more hydrophilic flavonoids, such as kaempferol, form hydrogen bonds with polar lipid head groups, compromising membrane integrity [108]. Among individual compounds, epigallocatechin gallate (EGCG) has been extensively studied and exerts similar overall antibacterial effects on both Gram-positive and Gram-negative bacteria, namely cell wall and membrane disruption leading to leakage, metabolic interference, and cell death. However, the specific mechanism differs slightly due to structural differences in the bacterial envelopes. In Gram-positive bacteria (e.g., *S. aureus*), EGCG primarily binds directly to peptidoglycan, weakening the thick cell wall and reducing osmotic stability, while in Gram-negative bacteria (e.g., *E. coli*), EGCG targets the outer membrane, interacting with porins and inducing oxidative stress, leading to membrane damage [7].

### 5.3. Interaction with Enzymes, Microbial Proteins, and Nucleic Acids

Polyphenols can also interfere with intracellular processes (Figure 2). The multiple hydroxyl groups attached to the hydrophobic benzene ring of polyphenols allow them to interact with proteins, enzymes, and nucleic acids (DNA and RNA) through amino, carboxyl, or hydrophobic interactions, thereby inhibiting enzyme activity, disrupting microbial metabolism, replication, and gene expression [7]. The bacterial DNA gyrase is an enzyme essential for DNA replication in prokaryotes (e.g., *Escherichia coli*). Several bioactive compounds have been shown to bind the β-subunit of DNA gyrase, blocking its ATP-binding site and inhibiting enzyme activity in a dose-dependent manner. This inhibition interferes with DNA replication and cell division, ultimately leading to growth arrest and antibacterial effects [111]. Several natural products bind bacterial targets in unique ways that can overcome resistance, whereas synthetic compounds (e.g., spiropyrimidinetriones, fluoro-quinolones), though highly potent, may cause off-target effects in human cells [112]. For instance, aminocoumarins, flavone derivatives, cyclothialidines, and green-tea catechin derivatives (EGCG, epicatechin-3-gallate, epigallocatechin, quercetin) inhibit bacterial DNA gyrase by targeting either one of its subunits. These compounds demonstrated promising activity against resistant bacterial strains while exhibiting lower toxicity toward eukaryotic cells compared to many synthetic antibiotics [112,113]. Dihydrofolate reductase (DHFR) is a key enzyme that converts dihydrofolate into tetrahydrofolate (THF), a cofactor required for one-carbon transfer reactions involved in the synthesis of purines and thymidylate, building blocks of DNA and RNA [114]. A study has shown that the green-tea polyphenol (-)-epigallocatechin-gallate (EGCG) directly inhibits DHFR at concentrations found in the plasma of tea drinkers (0.1–1.0 µM) [115]. Aslan et Adem [116] showed that naringin and ferulic acid exhibited strong inhibitory effects on enzyme activity, greater than most drugs except levofloxacin, while syringic acid, though the weakest inhibition among the natural products, still outperformed most synthetic drugs.

### 5.4. Induction of Oxidative Stress

Several plant-derived compounds exhibit strong antibacterial activity through their ability to induce oxidative stress within the bacterial cytoplasm (Figure 2). Reactive oxygen species (ROS) are naturally generated during aerobic metabolism and have shown strong antibacterial activity in vitro and in vivo against a broad range of Gram-positive and Gram-negative bacteria. Under physiological conditions, cellular antioxidant systems regulate ROS levels, but when ROS accumulate, they cause oxidative stress, leading to damage to DNA, proteins, and lipids [111,117]. Polyphenols disrupt cell membranes and walls, interfere with metabolic processes like ATP synthesis and ion transport (Ca^++^ and K^+^), and cause mitochondrial damage through ROS, acting as antibacterial agents. These mechanisms include altering membrane permeability, generating oxidative stress, and inhibiting essential enzymes, which collectively inhibit growth [7]. Plant-derived compounds like catechins, ferulic acid, and their derivatives have been shown to trigger oxidative stress in microbes, further enhancing their antibacterial potential [118].

Allicin, produced by garlic (*Allium sativum*), oxidizes thiol groups in cysteine-containing proteins in a dose-dependent manner, causing disulfide stress that reduces the viability of *Staphylococcus aureus* and *Bacillus subtilis* [119]. Allium species are recognized as an important source of organosulfur compounds that induce oxidative and disulfide stress in bacteria. These sulfur-containing metabolites, such as, thiosulfinates, ajoenes, and various disulfides, have been extensively characterized for their reactivity with thiol-containing proteins, ultimately disrupting redox balance and promoting intracellular oxidative damage [120,121]. Recent reviews emphasize that Allium-derived organosulfur compounds possess broad antimicrobial activities through mechanisms involving ROS generation, membrane disruption, and interference with essential metabolic pathways [122]. Sathiya Deepika et al. [123] demonstrated that rutin, as well as a rutin–gentamicin combination, inhibited biofilm formation in *P. aeruginosa* by inducing ROS, which triggered oxidative stress, disrupted the cell wall, and ultimately inhibited bacterial growth [123]. The number and position of hydroxyl groups, catechol B-ring presence, and C2-C3 double bond collectively determine whether a flavonoid functions as a direct antioxidant, a ROS-suppressing metal chelator, or a pro-oxidant agent capable of generating antibacterial ROS [124]. Quinones exhibit antibacterial activity through both bacteriostatic and bactericidal mechanisms, generating ROS via redox cycling between quinone and semiquinone forms, leading to intracellular oxidative stress, membrane damage, and ultimately cell death [125].

### 5.5. Inhibition of Quorum Sensing and Biofilm Formation

Quorum sensing (QS) is a cell-to-cell signaling mechanism through which bacteria release and detect extracellular signaling molecules (ESM), known as auto-inducers (AIs), to coordinate collective behaviors, such as virulence and biofilm development (Figure 2). Quorum sensing (QS) is triggered when autoinducers accumulate to a threshold concentration, bind to their cognate receptors, and activate the expression of genes controlling virulence factors and extracellular polymeric substance (EPS) production [126,127]. Several plant bioactive compounds exhibit antibacterial activity by targeting bacterial communication systems rather than growing directly, by inhibiting quorum sensing and biofilm development. Antibiofilm activity refers to the ability of a compound to prevent the establishment or maturation of bacterial biofilms, weakening the protective matrix and resistance to host defenses and antibiotics [128,129]. Bioactive compounds can disrupt QS, a process known as quorum quenching (QQ), through multiple mechanisms, including inhibition of autoinducer synthesis, enzymatic degradation, or sequestration of signaling molecules and competitive binding to QS receptors [129,130]. Reducing biofilm not only limits bacterial persistence but also makes the remaining cells more susceptible to conventional antibiotics [130]. This indirect mode of action makes them promising adjuncts to conventional antibiotics. Alkaloids, tannins, terpenes, and flavonoids are key phytochemicals that inhibit microbial growth and biofilm formation through diverse mechanisms. Compounds such as piperine and berberine interfere with QS pathways, suppress EPS production, and down-regulate genes involved in toxin synthesis and bacterial motility, thereby weakening biofilm structure and reducing microbial virulence [131]. Tannic acid, commonly found in gallnuts and tea, exerts its antibiofilm effects by chelating metal ions within the EPS matrix, which are essential for maintaining biofilm structural stability [132]. Similarly, naringenin suppresses the expression of biofilm-associated genes, thus compromising the structural cohesion of microbial communities [133]. Terpenes such as carvacrol, myrtenol, and thymol inhibit early biofilm development, disrupt mature biofilms, and weaken virulence factors in pathogens like *C. albicans*, *P. aeruginosa*, and *A. baumanni I* [134,135,136,137].

### 5.6. Efflux Pump and β-Lactamase Inhibition

Bacterial resistance to antibiotics often arises from intrinsic defense mechanisms such as efflux pumps and β-lactamase enzymes (Figure 2). Efflux pumps are transport proteins that actively expel toxic molecules, antibiotics, and signaling compounds from the bacterial cell, thereby reducing intracellular drug accumulation. Based on their energy sources, they are classified as primary pumps, which use ATP hydrolysis, or secondary pumps, which rely on electrochemical gradients like the proton motive force. These systems play a crucial role in bacterial survival under stress and are key contributors to multidrug resistance, making them promising targets for inhibition strategies [138,139].

Another major resistance mechanism is the enzymatic degradation of β-lactam antibiotics by β-lactamases. The primary mechanism of resistance to β-lactam antibiotics involves the bacterial production of β-lactamase enzymes, which break down the peptide bond in the four-membered β-lactam ring, thereby neutralizing the antibiotic’s effectiveness [140]. β-lactamases are grouped into four main classes (A–D): classes A, C, and D are serine hydrolases that use a serine residue at the active site, whereas class B, or metallo-β-lactamases, require zinc ions for catalysis [107].

Many plant metabolites, particularly phenolic compounds, can disrupt efflux pump function and β-lactamase activity, restoring bacterial susceptibility to antibiotics. Efflux pump inhibition may occur through several mechanisms, including interference with ATP production, dissipation of proton gradients, repression of efflux gene expression, or blockage of membrane transport proteins. By hindering these systems, phytochemicals enhance intracellular antibiotic concentration and promote bactericidal effects [108]. The antibacterial potential of plant metabolites largely depends on their molecular structure. Longer hydrophobic chains strengthen interactions with bacterial membranes, while excessive hydrophilicity can diminish antibacterial action [7].

## 6. Plant Extracts in Food Matrices as Preservatives

Food preservation is the process of ensuring that food quality and safety are maintained or often increased before reaching the consumer. Synthetic food preservatives such as nitrates, nitrites, benzoates, and sulfur dioxide are commonly used during storage to delay or prevent the spoilage of foodstuffs by inhibiting the growth of various foodborne microorganisms, such as bacteria and fungi that are both toxic to consumers and render the affected supplies inedible, causing significant loss of food products each year [141]. Modern studies have indicated that the extended use of such synthetic preservatives causes health concerns such as allergies, digestive disorders, and even cancers, which, coupled with the increased consumer demand for “clean-label” products, has turned the need for the discovery and application of natural alternatives imperative. Plant extracts, specifically those that are derived from edible plants, are considered natural antibacterial agents capable of solving this problem, often due to their high content in phenols, flavonoids, terpenes, tannins, and other bioactive compounds [141,142]. The data in Table 3 summarize application trials conducted in real food matrices, including meat, seafood and processed products and provide the foundation for discussing key case studies and trends in antibacterial efficacy. Application of plant extracts on processed meat products, such as various types of sausage (Frankfurter-type, Italian Cintra, Spanish Chorizo, and others), showed promising results. More specifically, the incorporation of *Urtica dioica* (stinging nettle) ethanolic extract at 500 ppm into the filling of Frankfurter-type sausages significantly reduced TVC compared with control samples, achieving a decrease of more than 1 log CFU·g^−1^ after 45 days of storage at 4 °C. Simultaneously, green tea and stinging nettle extracts inhibited the growth of yeasts and molds and completely inhibited the proliferation of coliforms [143]. Martínez et al. [144] reported similar findings during the fermentation of Spanish chorizo, where extracts of *Citrus sinensis*, *Rosmarinus officinalis* L. (rosemary), and *Malpighia emarginata* (acerola) were used as natural replacements for a commercial additive mix containing spices, salt, dextrose, lactose, milk protein, emulsifiers (triphosphates E-451, diphosphates E-450), flavor enhancer (monosodium glutamate E-621), preservative (sodium nitrate E-251), antioxidant (sodium ascorbate E-301), and coloring agent (carminic acid E-120). After 50 days of refrigerated storage, fermented sausage samples containing citrus, rosemary, and acerola extracts incorporated into the meat slurry exhibited a 24–60% reduction in total coliforms, complete inhibition of *Clostridium perfringens* (which was detected in the control), and a decrease in TVC by 0.16–0.23 log CFU·g^−1^. Notably, samples treated with citrus extract exhibited the strongest antibacterial effects, which the authors attributed to a potential synergistic interaction between the extract’s flavonoids and phenolic acids and the natural nitrate sources added to enhance flavor during fermentation. Although not conclusive, this study represents a promising step toward replacing synthetic additives traditionally used in Spanish chorizo with natural alternatives [144]. Citrus extracts are rich in flavonoids which function synergistically with terpenoids, displaying broad antimicrobial activity was they attack the lipid bilayer of the cellular membrane and disrupt it, causing cell lysis to take place. This mechanism slows down the biological spoilage, allowing the extract to be used as a preserving agent especially in meat products that tend to soil by present lipid oxidization and protein degradation [145]. In line with processed meat products, several studies have focused on the antibacterial effects of plant-derived extracts on minced meat. Since most extracts are used in an aqueous solution form, they can be easily incorporated and mixed in minced meat homogenously. Microbial Counts on minced pork meat and minced beef meat formulated into a patty, mixed with *Eugenia uniflora* L. (Pitanga) leaf and Salicornia extracts respectively and showed significant reduction in microbial counts, particularly during the end of the 18 and 15-day storage periods where treated samples displayed values of CFU·g^−1^ comparable to those achieved on burgers treated with Butylated hydroxytoluene (BHT) a synthetic antioxidant routinely used as food preservative and reduced late-stage fungal counts [146,147]. This highlights a clear trend of effective incorporation of plant extracts in meat products that results in decreased microbial activity and thus lengthening of their shelf-life without any significant drawbacks.

Fish and seafood are highly perishable food products due to their pH, fatty acids, high presence of free amino acids, and the presence of autolytic enzymes. For that reason, they require quick and efficient processing to avoid rapid deterioration. Most often, cold storage and refrigeration are applied, but the addition of antibacterial agents is often used as a supplementary tool for the improvement of food quality [148,149,150]. Recent studies on the antibacterial effects of plant extracts in fish and seafood increasingly report that the method of application, along with the type and concentration of the extract, strongly influences the magnitude of antibacterial activity. Miranda et al. [149] applied ethanolic extracts of *Chenopodium quinoa* Willd. directly to the ice used for refrigerating Atlantic chub mackerel. The high phenolic content of these extracts effectively inhibited microbial activity, as evidenced by the stable pH of the treated fish and the significantly lower trimethylamine-nitrogen (TMA-N·kg^−1^ flesh) values compared with the control. These results indicate that lipolytic bacteria, typically responsible for mackerel spoilage, remained active in the control samples but were markedly inhibited in those treated with 0.20 g·L^−1^ of the extract [149]. Summer savory leaf extract (SHE), incorporated into a carboxymethyl-cellulose (CMC) coating and applied by dipping onto fillets of *Lethrinus nebulosus* (spangled emperor), inhibited the growth of total bacteria and psychrotrophic bacteria. Total bacterial counts remained below the acceptable limit of 10^6^ CFU·g^−1^ established by the International Commission on Microbiological Specifications for Foods (ICMSF) [151] for fresh and frozen fish. A significant difference was observed between treatments with 1.5% and 0.5% extract, with the higher concentration consistently exhibiting greater antibacterial efficacy [152]. Mazandrani et al. [148] used liposomes to encapsulate ethanolic fennel extracts and then dipped silver carp fillets in a liposome-rich solution. Even at a low concentration (0.3%) and without encapsulation, fennel extract reduced the growth of foodborne bacteria by approximately 2 log CFU compared with untreated controls. Consequently, TVC in treated samples remained below the acceptable limit for fifteen days, whereas untreated fillets exceeded this limit by day six. Although encapsulation did not enhance antibacterial efficacy, the liposomal carrier appeared to protect phenolic compounds from degradation, resulting in the lowest peroxide (PV) and TBARS values among all treatments. In the study of Olatunde et al. [153], ethanolic extracts of *Morinda citrifolia* L. (Noni) leaves in two forms, with and without chlorophyll removal, were evaluated. Both extracts were applied to stripped-catfish slices, with the dechlorophyllized extract consistently performing better, reducing TVC and psychrotrophic bacteria by ≥2 log CFU·g^−1^ over nine days. Untreated catfish slices exceeded acceptable bacterial limits by day six, whereas treated samples remained safe for up to nine days (without chlorophyll removal) and twelve days (with chlorophyll removal). This extended shelf life is attributed primarily to flavonoid compounds, such as kaempferol and quercetin, which were preserved during chlorophyll removal and present at higher concentrations compared with the non-reduced extract [153].

High-throughput Illumina-MiSeq sequencing of container-cultured snakehead (*Channa argus*) fillets, correlated with TVC counts over 11 days, revealed that grape seed extract (GSE) delayed growth of spoilage bacteria for five days, extending fillet shelf life and suppressing overall bacterial proliferation. Initially, the microbiome of the fillet was diverse, dominated by *Qipengyuania* (29.3%), *Aeromonas* (15.3%), *Kocuria* (14.4%), *Comamonas* (6.5%), and *Macrococcus* (4.8%). During storage, these groups declined as *Pseudomonas* increased, with the control dominated by *Pseudomonas* (93.9%) and *Aeromonas* (5.4%) by day eleven. In GSE-treated fillets, 99.4% of bacteria were *Pseudomonas*, reflecting near-complete inhibition of *Aeromonas*, a major spoilage organism. The antibacterial activity of GSE was attributed to phenolic acids, catechins, and proanthocyanidins, known to disrupt bacterial cell membranes [154]. These findings suggest that the extract’s high phenolic content induces cell death by disrupting the cytoplasmic membrane, damaging membrane proteins, and interfering with membrane-bound enzymes [155].

The importance of phenolic content was also addressed through the application of extracts derived from *Porphyra yezoensis* (laver), a type of red algae, on Pacific white shrimp (*Litopenaeus vannamei*), a highly perishable crustacean that is valued for its high nutritional value and widely consumed. Shrimp samples were dip treated with a polyphenolic extract (PP), a polysaccharide-rich extract (PS), and a combination of the two (PP + PS). TVC results showed that while the control group passed the acceptable limit for seafood (10^6^ CFU·g^−1^) on day 4, the treated samples did not until day 8, demonstrating robust antibacterial control. While the exact mechanism of bacterial inhibition is unknown, the authors suggest that PS extracts present antibacterial action as the polysaccharides could potentially act like an edible film, limiting oxygen access to the food’s surface, which in turn could function as an explanation for the improved inhibitory activity of the synergistic PP+PS system [156].

The application of plant extracts to fruits, vegetables, and dairy products is faced with a different profile of spoilage, often driven by yeasts, molds, and specific bacteria that are relevant in high-moisture environments. Fresh-cut fruits are highly susceptible to spoilage, requiring effective treatments that target high-risk pathogens. Tests conducted on citrus fruits aimed to inhibit the growth of pathogenic fungi *Penicillium digitatum* (green mold) and *P. italicum* (blue mold) through the use of pomegranate peel extract (PPE). Just 100 ppm of PPE was enough to inhibit spore germination of the targeted fungi. Furthermore, “Satsuma” mandarins, artificially wounded to simulate post-harvest conditions, were treated with the PPE through dipping, which resulted in a mitigation of the wound infection and simultaneous minimization of lesion diameter [157]. Experiments conducted in minimally processed peach slices, dipped in the Mediterranean seagrass *Posidonia oceanica* (PO) and Green Tea (GT) extracts, yielded moderate inhibition of spoilage flora (TAC, *Pseudomonas*, yeasts, and molds) across a five-day period of storage [158].

In dairy products, the application of plant-based extracts aims to combat high-moisture, low-pH environments that are often breeding grounds for pathogenic microorganisms, such as *Listeria monocytogenes* and various members of the *Salmonella* genus. In quark cheese, a fermented milk product, UAE and Dynamic Maceration (DM) extracts of *Strawberry tree* (*Arbutus unedo* L.) showed promising results in inhibiting the growth of molds, yeasts, and Total Aerobic Mesophiles (TAM), outperforming even the synthetic preservative potassium sorbate in certain cases. DM extract appeared to consistently perform better than UAE extracts, especially against aerobic mesophiles [159]. In liquid dairy products, several extracts were applied to raw cow’s milk with *Rhus coriaria* (Sumac) fruits having the most efficient antibacterial activity as they reported a value of 4.7 × 10^3^ CFU·mL^−1^ compared to the control sample’s 9.2 × 10^8^ CFU·mL^−1^ and a total eradication of coliforms after a six-hour incubation period at 25 °C [160].

Usage of edible plant extracts on food products to stabilize their qualities and extend their shelf-life without the adverse effects of synthetic preservatives has shown promising results during the last decade, with polyphenol-rich, ethanolic extracts often performing at the same level as widely used artificial preservation agents. Patterns in the behavior and action mechanisms of these antibacterial agents, however, are only now beginning to emerge, data on long-term stability of extracts within complex matrices, interactions with processing (heat, curing, fermentation), and kinetics of release from coatings/encapsulates are limited. Additionally, modern experimental trials have shifted their focus towards more modern methods of incorporating antimicrobial agents within food matrixes, making use of novel technologies such as encapsulation of agents withing various formations (nanomatrixes, lipids, coatings). The distinct lack of a study that aims to bridge the gap between traditional incorporation and modern approaches while also standardizing the testing procedures, prevents these methods from graduating from a lab environment to real-world applications.

Although edible plant extracts have demonstrated strong antibacterial efficacy in various food matrices, their incorporation into real food systems presents several formulation and sensory challenges that must be addressed to ensure industrial feasibility. Flavor masking remains a key concern, especially with phenolic-rich extracts such as rosemary, grape seed, and pomegranate peel, which can impart bitterness or herbal notes at higher concentrations, potentially affecting consumer acceptance [87,154,161]. Nanoencapsulation and dechlorophyllization have been employed to mitigate these effects, as seen in rosemary-treated beef [87] and noni-treated catfish, where sensory neutrality was preserved while maintaining microbial inhibition [153]. Stability is another critical factor, as bioactive compounds may degrade during processing or storage. Liposomal encapsulation of fennel extract in silver carp fillets improved oxidative stability and extended shelf life, suggesting that carrier systems can protect phenolics from oxidation and volatilization [148]. Interactions with food matrices also influence efficacy, for instance in pork burgers, *Eugenia uniflora* extract showed reduced antibacterial activity in later storage stages, likely due to protein-polyphenol binding that limited extract diffusion [146]. Conversely, in dairy systems such as quark cheese, *Arbutus unedo* extracts performed better than potassium sorbate, possibly due to favorable pH and moisture conditions that enhanced extract activity [159]. These findings highlight the need to customize the composition, dosage and delivery strategy of plant extracts according to the specific characteristics of each food matrix, ensuring optimal antimicrobial efficacy while preserving desirable sensory attributes and maintaining physicochemical stability.

**Table 3:** Application trials of edible plant extracts obtained by green extraction methods in real food matrices (meat, seafood, dairy and ready-to-eat products).

Plant (Part)	Food Matrix	Application (Including Extraction Method)	Target Microorganisms	Main Result	Ref.
*Eugenia uniflora* L. (Pitanga) (Leaves)	Pork Burgers	Hydroethanolic (40:60 H_2_O/EtOH) UAE/Stirring (80 °C). Mixed with minced meat (250–1000 mg/kg)	TVC, LAB, *Pseudomonas* spp.	Significantly ↓ microbial counts, mainly at the end of 18 d shelf-life	[146]
*Cymbopogon citratus* (Lemongrass) (Leaves)	Cooked and Shredded Chicken Breast	Hydro-ethanolic (95% EtOH) extract. Added to meat (1% *v*/*w*)	TCC, *Staphylococci*, *Salmonella* sp.	*Staph*, *Salmonella*, and Coliforms Not Detected at 45 °C during 60 d storage	[162]
*Rosmarinus officinalis* L. (Rosemary) (Aerial parts)	Beef meat	Nano-encapsulated (Soybean Protein Isolate/Basil Gum). Immersion (60 min). 1600 ppm	TVC	1600 ppm extract maintained TVC < 7 logCFU g^−1^ until d21	[87]
*Olea europaea* (Olive)/*Urtica dioica* (Stinging Nettle)/*Camellia sinensis* (Green Tea) (Leaves)	Frankfurter type sausage	EtOH (95%) extraction. Incorporated at 500 ppm before cooking/stuffing	TVC, TCC, yeasts/molds	TVC reduced (Stinging Nettle extracts best). Coliforms Not Detected	[143]
*Citrus reticulata/Citrus sinensis/Citrus bigarradia/Citrus macrocarpa* (Citrus) (Peel)	Beef tenderloin	Hydrodistilled extracts (100 °C, 6 h). Boiling in 50 g/L of the corresponding Citrus peel extract.	TBC, TAC	Significant ↓ microbial counts especially at d8. *Citrus reticulata* performed best	[145]
*Castanea sativa* (Chestnut) (Nut)/*Vitis vinifera* (Grape) (Seeds)	Italian Cinta Senese dry-fermented sausages	CHE and GSE mixed with tocopherol/hydroxytyrosol, replacing sodium nitrate in sausage	TVC, Prokaryotic communities (Illumina MiSeq)	Spoilage *Photobacterium* genus >30x lower. CHE/GSE. Extracts did not alter the prokaryotic community	[163]
*Citrus sinesis* L. (Orange)/*Rosmarinus officinalis* L. (Rosemary)/*Malpighia emarginata* (Acerola)	Spanish Chorizo (Fermented)	Combined with natural nitrate sources/spices. Mixed with meat paste	TVC, TCC, *Clostridium perfringens*	No growth of *C. perfringens*. Citric extracts showed the lowest viable growth	[144]
*Pistacia vera* (Pistachio) (Hull)	Fermented beef sausage	Water extract (1:15, 8 h stirring). Added to meat dough (500, 750, 1000 ppm)	TVC, LAB, staphylococci, yeasts & molds	The highest dose (1000 ppm) showed the lowest TVC (d28)	[164]
*Coriandrum sativum* L. (Coriander) (Seed)	Poultry meatballs	Commercial extract. Added to minced meat (200 ppm and 500 ppm)	TAM	500 ppm inhibited aerobic growth after d6. 200 ppm had no influence	[165]
*Prunus cerasus* (Cherry) (Leaves)/*Ribes nigrum* (Blackcurrant) (Leaves)	Pork meat sausages	Water extracts. Added to meat (0.5–1.0 g/100 g) before stuffing	TMC, PTC, LAB, *Brochothrix*, *Pseudomonas*, *Enterobacteriaceae*	Mesophiles, psychrotrophs, LAB, *Brochothrix* ↓ after 14 d	[166]
*Castanea sativa* (Chestnut) (Leaves, Bur, Hull)	Beef patties	Leaf: Acidified water. (25 °C, 90 mins) Bur and Hull: Water in pressurized reactor (220 °C–Bur/130 °C–Hull), non-ionic polymer resins, 96%EtOH (35 °C)	TVC, Psychotropic bacteria, LAB, *Pseudomonas* spp.	Leaf extract showed the lowest CFU for TVC, Psych., *Pseudomonas*. Bur extract showed higher CFU than the control	[167]
*Salicornia perennans* (Glassworts) (Leaves)	Beef patties	UAE EtOH (70%) extraction. Mixed with minced meat.	TAM, TPC, yeasts & molds	1.0–1.5% extract significantly ↓ all microbial counts for 15 d	[147]
*Punica granatum* L. (Pomegranate) (Peel)/*Cynara cardunculus* L. (Artichoke) (Leaves)	Sardine Fillets	Water extract (95 °C). Marinated in 5% solution (72 h) with 4% Acetic Acid/10% NaCl	TVC, LAB, TCC, *S. aureus*	LAB growth inhibited post-marination; Significant ↓ TVC/Coliforms after d30 (Pomegranate best)	[161]
*Cuminum Cyminum* L. (Cumin) (Seeds)/*Mentha Longifolia* L. (Wild mint) (Leaves)	Rainbow Trout fillets	EtOH extracts. Dipped in 3.0% and 6.0% (*w*/*v*) aqueous solution	TVC, PTC, *E. coli*, *S. Aureus*, *L. monocytogenes*	Mint showed lower TVC/PTC than Cumin. All treated samples < limit until d18	[168]
*Solanum lycopersicum* (Tomato) (Plant)	Sierra fillets	EtOH/Acetic acid (95:5 *v*/*v*) extract. Dipped in 0.3% TPE or TPE-C (Chitosan coating)	TAM	TPE/TPE-C delayed bacterial growth for 15 d	[169]
*Gracilaria sp*. (Red Algae) (Plant)	Pangas Fillets	EtOH (99%) extraction. Dipped in 3 concs (2% best) for 10 min	APC, Pychrophillic bacteria, *Enterobacteriacease*, *Staphylococcus*	2% GE allowed 6 more days of storage	[170]
*Allium ascalonicum* L. (Shallot) (Fruit)/Trachyspermum ammi (Ajwain) (Seed)	Rainbow trout (semi-fried)	EtOH (85%) extraction. Mixed into edible coating (1.5%/3% *v*/*v*). Semi-fried	TVC, PTC, total aerobic count, *Pseudomonas* spp.	3% Ajwain extract extended storage up to 9 more days. Ajwain consistently lower counts than Shallot	[171]
*Satureja hortensis* (Summer savory) (Leaves and stems)	Spangled Emperor fillets	EtOH (80%) extract combined with CMC coating. Immersion (10 min)	TVC, PTC	CMC + 1% SHE and CMC + 1.5% SHE extended storage life by 3 days	[152]
*Chenopodium quinoa* (Quinoa) (Grain)	Atlantic Chub Mackerel	EtOH (80%*v*/*v*) extract. Used as ice flakes for chill storage	Lipolytic bacteria	Quinoa extract inhibited the growth of lipolytic bacteria proportional to concentration	[149]
*Punica granatum* (Pomegranate) (Peel)	Nile tilapia fillets	EtOH (70%) extract. Added to Chitosan coating (1% PPE). Immersion (1 min)	TVC, Psychrotrophs, Yeasts/Molds, Coliforms, *E. coli*, *Salmonella* spp.	Complete Inhibition of most groups (30 d). TVC; ↓ 73.2%; Psychrotrophs ↓53.9%.	[172]
*Mentha arvensis* (Mint) (Leaves)/*Citrus aurantium* (Citrus) (Peel)	Indian mackerel	EtOH (60%) extraction. Dipped in 0.5% (Mint) or 1% (Citrus) solution (30 min)	APC	Mint extract extended acceptable limit by 5 days (to d16)	[173]
*Stevia rebaudiana* Bertoni (stevia) (Leaves)	Catla fillets	EtOH (80%*v*/*v*) extract. Dipped to form edible coating (2% best)	APC, PBC, LAB, *Enterobacteriaceae*, *Staphyloccocus*	2% Stevia leaf extract extended shelf life for 8 more days	[174]
*Allium paradoxum* (Few-flowered leek) (Leaves)/*Eryngium caucasicum* (Leaves)	Silver carp fillets	EtOH (80%) extraction. Dipped in 2% and 5% concs (30 min)	TVC, PTC	Extended storage life by 3 to 9 days. *A. paradoxum* (4%) showed lowest values	[175]
*Foeniculum vulgare* (Fennel) (Plant)	Silver carp fillets	EtOH extract. Used alone and liposome-encapsulated. Dipped for 15 min	TVC, TPC	Encapsulated extracts presented the best results by day 15	[148]
*Morinda citrifolia* (Noni) (Leaves)	Striped Catfish slices	EtOH (70%*v*/*v*) extract. Used as-is and dechlorophyllized. Mixed with fish slices	PBC, TVC	Extracts doubled storage period. DE consistently lower counts	[153]
*Punica granatum* (Pomegranate) (Fruit)/*Rosmarinus officinalis* L. (Rosemary) (De-oiled Leaf)/Olea europaea (Olive) (Leaf/Fruit)	Fish patties	Hydroethanolic extracts mixed into fish paste	TVC, TCC, *E. Coli*, *S. Aureus*, *L. monocytogenes*	HYT-F/HYT-L (Olive) had lowest TVC (d11). Pomegranate/Rosemary diterpene extracts (NOS/NOVS/RA) had lowest TCC	[176]
*Posidonia Oceanica* (Leaves)	Peach slices (Fresh-cut)	50% EtOH extraction. Dipped in 2% *w*/*v* solution (3 min)	TAC, *Pseudomonas*, Yeasts & molds, *Enterobacteriaceae*	Significant ↓ in yeasts/molds (3 d), *Pseudomonas*, and TAC. No change in *Enterobacteriaceae*	[158]
*Vitis vinifera* (Grape) (Seeds)	Snakehead fillets	60% EtOH UAE. Immersed in GSE solution (0.52 mg GAE/mL) for 20 min	TVC, Microbiota (Illumina MiSeq)	Slower TVC growth (extended shelf-life by 4 days). Inhibited *Aeromonas* growth	[154]
*Porphyra yezoensis* (Red Algae, Nori) (Plant)	Pacific white shrimp	Polyphenolic: EtOH (70%) & UAE. Polysaccharide: Water & UAE. Dipping (5 g/L extract for 60 min)	TVC	Extract mixture slowed TVC increase, reaching limit 4 days after control sample	[156]
*Punica granatum* (Pomegranate) (Peels)	Wounded “Satsuma” Mandarins	PPE (60% EtOH w/citric acid). Dipped for 1 min (Curative/Preventative)	*Penicillium italicum*, *Penicillium digitatum* (Molds)	Increased conc. showed an 80–90% ↓ infection rate and reduced lesion diameter	[157]
*Arbutus unedo* L. (Strawberry tree) (Leaf)	Quark cheese	Ultrasonic Assisted (UAE) and Dynamic Maceration (DM) extracts. Mixed into cheese (0.1 g/100 g)	TAM, *Enterobacteriaceae*, Molds, Yeasts, LAB, *S. aureus*	DM extracts performed better than UAE/Sorbate, significantly ↓ Molds, Yeast, TAM	[159]
*Rhus coriaria* (Sumac) (Fruits)/*Tamarindus indica* (Tamarind) (Pods)/*Rosmarinus officinalis* L. (Rosemary) (Aerial Parts)/*Hibiscus sabdariffa* (Roselle) (Red calyces)/*Citrus limon* (Lemon) (Fruits)	Raw Cow milk	EtOH (80%) extraction. Added at 3000 ppm to milk	TVC, TCC	Sumac most effective in ↓ total bacteria. Coliforms Not Detected in any treated sample	[160]

TVC: Total Viable Count; LAB: Lactic Acid Bacteria; TPC: Total Psychrotrophic Count; PTC: Phychrophilic Bacterial Count; TAM: Total Aerobic Mesophiles; TCC: Total Coliform Count; TMC: Total Mesophilic Count; PBC: Psychrophilic Bacteria Count; TAC: Total Aerobic Count; APC: Aerobic Plate Count.

## 7. Applications in Industry & Challenges

Although the antibacterial and antioxidant potential of plant-derived extracts has been widely reported, and their mechanisms of action have been partially elucidated, their translation from laboratory research to industrial-scale food preservation remains limited. Nevertheless, several commercial examples illustrate the successful implementation of plant extracts as natural preservatives and antioxidants. Herbalox^®^ (Kalsec) [177], StabilEnhance OSR4 [178] (Givaudan/Naturex), and OxiKan^®^ (Mane Kancor) [179] are rosemary extracts used to prevent lipid oxidation in meat products, edible oils, snacks, and baked goods. FORTIUM^®^ R [180], NaturFORT™ [181], OLESSENCE™ [182] (Kemin) are rosemary, green tea, and olive extracts, respectively, applied in processed meats, sauces, and dressings to extend shelf life and replace synthetic antioxidants. Despite these industrial examples, few widely recognized food brands explicitly label plant extracts as preservatives. This limited visibility can be attributed to several factors: (i) marketing strategies, as many producers include these extracts under “natural flavors” or “rosemary extract” without emphasizing their preservative role; (ii) regulatory and labeling complexities, since plant extracts with antioxidant activity are often classified as flavorings or natural additives rather than preservatives; (iii) cost and sensory considerations, as effective concentrations may alter flavor, aroma, or color; and (iv) shelf-life constraints, as long-term microbial stability still frequently requires synthetic preservatives or advanced packaging solutions. Therefore, although the efficacy of plant extracts is well supported and their use is gradually expanding, there remains a clear gap between research advances and their full industrial integration.

## 8. Future Perspectives

Looking forward, the current limitations and conventional extraction approaches highlight several important perspectives for future research and industrial applications. Most of the commercially available extracts described above are obtained via conventional solvent-based or hydroalcoholic extraction methods, which, while effective, may involve higher energy consumption, solvent use, and potential environmental impact. This underscores the need for exploring greener, more sustainable extraction technologies that could enhance yield, selectivity, and stability of the bioactive compounds while reducing ecological footprint. Although extensive research has been conducted on the antibacterial activity of plant extracts obtained through conventional methods, studies employing green extraction techniques remain comparatively limited. Similarly, while the antibacterial potential of individual bioactive compounds has been well characterized, there is a relative lack of research on the complex interactions and overall antibacterial efficacy of whole extracts, where synergistic or antagonistic effects may influence biological performance. Furthermore, regulatory perspectives on using plant extracts as natural preservatives are highly structured, requiring robust safety and efficacy assessments before a product can be authorized for use. In the European Union, a strict pre-market approval system necessitates a full safety review by the European Food Safety Authority (EFSA) for inclusion on an authorized “Union List” as per Regulation (EC) No 1333/2008 [183], regardless of the ingredient’s “natural” origin. Conversely, the United States employs a system where many traditional plant-based ingredients can be classified as “Generally Recognized as Safe” (GRAS), allowing for their use without explicit FDA pre-market approval, provided the manufacturer has sufficient scientific data to support safety (FDA) [184]. Across both jurisdictions, key challenges include standardizing the highly variable composition of plant extracts to ensure consistent performance and safety, and providing clear scientific evidence that they are effective alternatives to synthetic preservatives under real-world food conditions [185,186].

To support future progress, research and development should focus on the following priority areas:

• Optimizing green extraction methods to improve yield, selectivity, scalability, and cost efficiency.
• Standardizing analytical and extraction protocols to ensure consistent identification, quantification, and comparison of active compounds across studies and species.
• Investigating synergistic and antagonistic interactions within whole extracts and assessing their antibacterial and antioxidant performance in real food matrices.
• Conducting application-focused studies including sensory evaluation and shelf-life testing to support commercial feasibility.
• Developing multifunctional or synergistic extract combinations to enhance antimicrobial spectra and reduce required dosages.
• Advancing industrial upscaling strategies, including solvent recovery, process reproducibility, and energy-efficient operation.
• Establishing regulatory frameworks and clear labeling guidelines specific to green-extracted plant preservatives.
• Improving consumer acceptance and communication, emphasizing natural, sustainable, and clean-label benefits.

Together, these efforts will be essential to translating promising laboratory findings into commercially viable, sustainable food-preservation solutions.

## 9. Conclusions

Edible plant extracts represent a sustainable source of antibacterial agents offering promising solutions to the rising concerns over synthetic preservatives and antibiotic resistance. Green extraction methods have emerged as an effective approach to enhance the antibacterial activity of those extracts, while minimizing environmental impact. Various mechanisms have been proposed to explain their antibacterial activity. Although numerous studies have investigated the antibacterial properties of the plants extracts obtained through green extraction methods, their incorporation in food systems as preservatives remains limited compared to extracts produced by conventional methods. Nevertheless, polyphenol-rich extracts have demonstrated strong efficacy in prolonging shelf life and maintaining the microbiological quality of diverse food products, such as meat, seafood, dairy, and fruit products. A number of challenges still hinder large-scale industrial adoption. Extract composition can vary widely due to differences in raw materials and extraction parameters, complicating standardization and reproducibility. Regulatory frameworks for green-extracted plant-based preservatives are not yet standardized, creating uncertainty for producers and limiting market entry. Additionally, sensory effects, particularly changes in flavor, color, or aroma, may restrict the use of plant extracts at concentrations required for microbial inhibition. Consumer expectations for natural, clean-label products also introduce communication challenges, requiring clear and trustworthy labeling. Moving forward, progress in regulatory alignment, technological optimization, and consumer acceptance strategies will be essential to translate laboratory findings into commercially viable, food-grade antibacterial solutions. With continued efforts in these areas, green-extracted plant preservatives have strong potential to become mainstream tools in sustainable food preservation.

## Figures and Tables

**Table 1:** Comparative evaluation of green extraction technologies.

Method	Extraction Efficiency	Yield	Solvent Use	Energy Consumption	Key Limitations	Industrial Scalability	Future Directions	Regulatory Acceptance
NADES	polar (phenolics)/some terpenoids	very high	very low (no toxic solvents)	low	high viscosity; solvent recovery; regulatory gaps	emerging (strong potential but not yet widely adopted)	developing scalable and cost-effective recovery and purification methods; toxicological studies	emerging/Low
UAE	polar (phenolics)/ non-polar (terpenes)	high	low–moderate (aqueous/ ethanolic)	low	localized heat spots; compounds degradation	high (economical & scalable)	real-time monitoring for efficiency and energy use optimization; focus on hybrid systems	high widely accepted established safety guidelines for ultrasound exposure
MAE	polar (phenolics)	very high	low (polar solvents needed)	moderate	expensive equipment; limited microwave penetration depth in large volumes	moderate (scaling requires specialized reactors)	optimization of continuous-flow reactors with lower frequencies	moderate accepted for specific applications
EAE	bound phenolics	high	low (water-based)	low	enzyme cost; variability; potential enzyme deactivation	moderate (cost-limited)	creation of enzymes with enhanced stability and activity for specific industrial conditions	Very high well-established and accepted technology in food processing and pharmaceuticals
SFE (CO_2_)	non-polar (terpenes/ terpenoids)	very high	very low	moderate–high (pressurized CO_2_)	high capital cost; trained operators	moderate–high(used in food & pharma)	exploring SFE as a hybrid method for targeted fractionation of extracts	very high well-established, non-toxic, widely used in food and pharmaceutical industries
HHP	polar (phenolics)	high	moderate (aqueous/ ethanolic)	low-moderate	batch process; high capital cost	commercially viable and highly scalable	development of continuous systems to improve throughput	high well-regulated for safety and efficacy in food industry; growing acceptance in pharmaceutical industry

**Table 2:** Antibacterial activity of plant extracts against microorganisms, their extraction methods, the antibacterial bioassays, and the main antibacterial mechanism of action from the literature.

Plant *Official Name*, Common Name, Plant Part	Extraction Method & Conditions SLR (g/mL); T (min)	Antibacterial Assay	Microorganism	Antibacterial Activity		Ref.
				Inhibition Zone (mm)	MIC	
*Hibiscus sabdariffa*, Roselle	UAE EtOH:Water (90:10 *v*/*v*) 53 kHz, 1:18, 30 min	Agar well diffusion	*E. coli* *B. cereus*	21.1 22.2	-	[64]
*Rosmarinus* *officinalis*, Rosemary			*E. coli* *B. cereus*	17.4 16.7		
*Syzygium aromaticum*, Clove			*E. coli* *B. cereus*	21.1 19.8		
*Thymus vulgaris*, Thyme			*E. coli* *B. cereus*	15.9 17.3		
*Vaccinium vitis-idaea*, Lingonberry, leaves	UAE EtOH:Water:Acetic acid (70:30:1 *v*/*v*/*v*), 1:10, 30 min	Broth microdilution	*B. cereus* *S. enterica sv.* Typhimurium	-	100 71	[85]
*Ribes rubrum* var. alba, White currant, leaves			*B. cereus* *S. enterica sv.* Typhimurium		90 78	
*Crataegus* spp., Hawthorn, leaves			*B. cereus* *S. enterica sv.* Typhimurium		100 86	
*Hippophae rhamnoides*, Sea buckthorn, leaves			*B. cereus* *S. enterica sv.* Typhimurium		100 100	
*Amelanchier alnifolia*, Saskatoon, leaves			*B. cereus* *S. enterica sv.* Typhimurium		890	
*Rubus idaeus*, Raspberry leaves			*B. cereus* *S. enterica sv.* Typhimurium		96 81 ^3^	
*Hippophae rhamnoides*, sea buckthorn, leaves	UAE EtOH:Water (50:50 *v*/*v*) 40 kHz, 1:5 *w*/*v*, 1 h	Broth microdilution	*S. aureus* *B. cereus* *P. aeruginosa*	-	6.20 12.5 6.20 ^1^	[86]
*Rosmarinus officinalis* L., Rosemary leaves	UAE EtOH:Water (50:50 *v*/*v*) 20 kHz, 1:10, 20 min	Broth microdilution	*S. aureus* *P. aeruginosa* *E. coli*	-	0.140 0.360 0.340 ^1^	[87]
*Chaenomeles japonica* (Thunb.) Lindl. ex Spach, Japanese quince, fruits	UAE EtOH:Water (50:50 *v*/*v*) 480 W, 1:20, 20 min		*B. subtilis* *E. faecalis* *S. aureus* *E. coli*	21.7 30.7 18.7 19.6	-	[88]
*Rosa canina* L., Dog rose, pseudofruit	UAE Methanol, 1:6, 45 min	Broth microdilution	*P. aeruginosa* *E. coli*	-	0.256 >0.512 ^1^	[89]
*Punica granatum* L., Pomegranate, Peels	UAE EtOH:Water (30:70 *v*/*v*) with 1% formic acid, 1:10, 30 min	Broth microdilution	*A. baumannii* *S. aureus* *P. aeruginosa* *E. coli*	-	3.2 0.8 6.4 12.8 ^1^	[90]
*Punica granatum* L., Pomegranate, Peels	MAE 1500 W, 1:30, 10 min	Agar disk diffusion	*P. aeruginosa* *E. coli* *S. aureus*	15 19 22	-	[91]
*Punica granatum* L., Pomegranate, Peels	MAE 900 W, 1:8, 8 min	Broth microdilution	*S. aureus* *E. coli* *P. aeruginosa* *Proteus mirabilis*	-	12.5 25 25 50 ^2^	[92]
*Piper betle* L., Betel, leaves	MAE 239.6 W, 1:22, 1.6 min	Agar well diffusion	*B. pumilus* *B. cereus* *K. pneumoniae* *E. coli*	3.2 2.6 2.5 2.8	-	[93]
*Levisticum officinale*, Lovage, leaves	MAE 53% EtOH, 800 W	Broth microdilution	*S. aureus* *E. coli*	-	13.5 13.5 ^1^	[94]
*Origanum vulgare*, Oregano, leaves	MAE 49% EtOH, 160 W				3.06 13.5 ^1^	
*Salvia officinalis*, Sage leaves	MAE 800 W, 8 min	Broth microdilution	*E. coli* *E. asburiae* *M. luteus* *B. subtilis*	-	27.50 56.65 5.69 18.43 ^4^	[95]
*Olea europaea* L., Olive, leaves	MAE Water, 800 W, 1:8, 10 min	Broth microdilution	*S. aureus* *S. enterica sv.* Typhimurium *E. coli* *L. monocytogenes*	-	2.5 40 40 30 ^1^	[96]
*Hippophae rhamnoides*, Sea buckthorn, leaves	EAE Viscozyme L and Cellulase 1% (*v*/*w*), 1:20 (*w*/*v*), 3.15 h	Agar well diffusion	*S. aureus*	4.17	-	[97]
*Rosa canina* L., Dog rose, pseudofruit	EAE Phosphate buffer pH 5.5, Cellic Ctec3 1% *v*/*v*, 1:16.67, 360 min	Broth microdilution	*E. coli*	-	80 ^3^	[98]
*Rosa canina* L., Dog rose, pseudofruit	EAE Phosphate buffer pH 5.5, Pectinex Ultra color 0.59% *v*/*v*, Viscoferm 0.51%, 1:16.67, 96 min	Broth microdilution	*E. coli*	-	55 ^3^	[99]
*Punica granatum* L., Pomegranate, Peels	HHP Water, 600 MPa, 1:62.5, 15 min	Agar well diffusion	*S. aureus* *B. cereus* *P. aeruginosa* *E. coli*	20 18 29 10	7.82 15.63 62.5 62.5 ^1^	[100]
*Origanum vulgare*, Oregano	SFE CO_2_ (purity 2.5%), 25 MPa	Broth microdilution	*S. aureus* *E. coli* *C. albicans*	-	0.147 0.728 0.311 ^1^	[101]
*Mentha piperita*, Peppermint, leaves	NADES Choline chloride: citric acid (1:1) + 30% water, 75:1, 30 min	Broth microdilution	*P. aeruginosa* *S. aureus* *E. coli* *S. enterica sv.* Typhimurium	-	0.39 0.39 0.78 0.78 ^3^	[102]
*Malva sylvestris* L., mallow, flower	NADES Choline chloride: citric acid (1:1) + 30% water, 1:13.3, 60 min	Agar well diffusion	*S. aureus* *E. coli* *P. aeruginosa* *B. cereus*	33.3 30.0 32.0 31.3	-	[103]

^1^ mg/mL, ^2^ μg/mL, ^3^ %, ^4^ μL/mL.

## Data Availability

No new data were created or analyzed in this study. Data sharing is not applicable to this article.

## Abbreviations

The following abbreviations are used in this manuscript

AMR	Antimicrobial Resistance
UAE	Ultrasound-Assisted Extraction
MAE	Microwave-Assisted Extraction
EAE	Enzyme-Assisted Extraction
NADES	Natural Deep Eutectic Solvents-Assisted Extraction
ILs	Ionic Liquids
DES	Deep Eutectic Solvents
HBA	Hydrogen Bond Acceptor
HBD	Hydrogen Bond Donor
SFE	Supercritical Fluid Extraction
HHP	High Hydrostatic Pressure
MIC	Minimum Inhibitory Concentration
MBC	Minimum Bactericidal Concentration
CFUs	Colony-Forming Units
TLC	Thin-Layer Chromatography
HPLC	High-Performance Liquid Chromatography
OPLC	Over-Pressured-Layer Chromatography
CV	Crystal Violet
cFDA	Carboxyfluorescein Diacetate
STC	Standard Plate Count
TVC	Total Viable Count
ACC	Aerobic Colony Count
AMC	Aerobic Mesophilic Count
CFU·g^−1^ or CFU·mL^−1^	Colony-Forming Units per gram or milliliter
FDA	Food and Drug Administration
NGS	Next-Generation Sequencing
PCR	Polymerase Chain Reaction
EGCG	Epigallocatechin Gallate
DHFR	Dihydrofolate Reductase
THF	Tetrahydrofolate
ROS	Reactive Oxygen Species
QS	Quorum Sensing
ESM	Extracellular Signaling Molecules
AIs	Auto-Inducers
EPS	Extracellular Polymeric Substance
QQ	Quorum Quenching
AHL	Acyl-homoserine lactones
ICMSF	International Commission on Microbiological Specifications for Foods
List of microorganisms and their abbreviations	
*A. baumannii*	*Acinetobacter baumannii*
*B. cereus*	*Bacillus cereus*
*B. pumilus*	*Bacillus pumilus*
*B. subtilis*	*Bacillus subtilis*
*C. albicans*	*Candida albicans*
*E. asburiae*	*Enterobacter asburiae*
*E. coli*	*Escherichia coli*
*E. faecalis*	*Enterococcus faecalis*
*K. pneumoniae*	*Klebsiella pneumoniae*
*L. innocua*	*Listeria innocua*
*L. monocytogenes*	*Listeria monocytogenes*
*M. luteus*	*Micrococcus luteus*
*M.R.S. aureus*	*Methicillin-Resistant Staphylococcus aureus* (*MRSA*)
*P. aeruginosa*	*Pseudomonas aeruginosa*
*P. mirabilis*	*Proteus mirabilis*
*S. aureus*	*Staphylococcus aureus*
*S. enterica*	*Salmonella enterica*
*S. enterica sv.* Typhimurium	*Salmonella enterica serovar* Typhimurium
*S.* Enteritidis	*Salmonella Enteritidis*
*S.* Typhimurium	*Salmonella Typhimurium* (*synonym for S. enterica sv.* Typhimurium)
*V. parahaemolyticus*	*Vibrio parahaemolyticus*
*Y. enterocolitica*	*Yersinia enterocolitica*
